# Pre‐Existing Nocturia Status Predicts Bladder Symptom Exacerbation Following COVID‐19 Vaccination in Women

**DOI:** 10.1002/kjm2.70177

**Published:** 2026-01-22

**Authors:** Hsien‐Che Ou, Hao‐Wei Chen, Yung‐Shun Juan, Yu‐Chen Chen

**Affiliations:** ^1^ Department of Urology Kaohsiung Medical University Hospital, Kaohsiung Medical University Kaohsiung Taiwan; ^2^ Department of Urology, School of Medicine, College of Medicine Kaohsiung Medical University Kaohsiung Taiwan; ^3^ Graduate Institute of Clinical Medicine, College of Medicine Kaohsiung Medical University Kaohsiung Taiwan; ^4^ Center for Big Data Research Kaohsiung Medical University Kaohsiung Taiwan; ^5^ Regenerative Medicine and Cell Therapy Research Center Kaohsiung Medical University Kaohsiung Taiwan

Bladder sensory symptoms following COVID‐19 vaccination remain underrecognized, particularly in women. Prior surveys have suggested transient storage lower urinary tract symptom (LUTS) changes after vaccination; however, these studies often pooled data across sexes and utilized overactive bladder‐oriented instruments that do not capture the “problem/bother” dimension central to interstitial cystitis/bladder pain syndrome (IC/BPS)‐like phenotypes [1, 2, 3]. We therefore evaluated whether COVID‐19 vaccination is associated with measurable changes in IC/BPS‐specific symptom and problem indices in women and sought clinical predictors of post‐vaccination deterioration.

We conducted a cross‐sectional, questionnaire‐based survey at a Taiwanese vaccination clinic from October 1, 2021 to January 31, 2022. Adult women who had received at least one COVID‐19 vaccine dose completed Chinese‐validated versions of the Interstitial Cystitis Symptom Index (ICSI) and Problem Index (ICPI) before and after vaccination [4]. General adverse events following Covid‐19 vaccination was also documented (Supplementary table 1). Participants with pregnancy or a physician‐diagnosed urinary tract infection in the preceding 3 months were excluded. The primary outcome was clinically meaningful deterioration, defined as any increase in ICPI total score after vaccination. Logistic regression was used to identify predictors including baseline nocturia and metabolic comorbidities, given prior links between metabolic risk and female LUTS [5] (Supplementary table 2 and 3).

A total of 595 women completed eligible surveys. Post‐vaccination, ICSI subscores for frequency, nocturia, urgency, and bladder pain increased significantly, with a modest but significant rise in ICSI total score. Similarly, ICPI subscores for frequency, nocturia, and urgency also increased (Supplementary table 4), though the total change in ICPI was small overall. Notably, 123 women (20.7%) experienced ICPI total score deterioration, indicating worsened patient‐perceived bother following vaccination. A small subset (5.2%) reported seeking medical attention for urinary complaints, consistent with real‐world signals that urologic adverse effects can occur but are generally uncommon [3].

Baseline nocturia appeared to stratify post‐vaccination trajectory. Women with pre‐existing nocturia exhibited significantly greater increases in ICPI total score than those without nocturia (Table 1). In multivariable analysis, pre‐existing nocturia (adjusted OR 3.53, 95% CI 2.29–5.21) and hyperlipidemia (adjusted OR 3.42, 95% CI 1.24–9.46) independently predicted ICPI deterioration.

**TABLE 1:** Comparison of bladder sensory symptoms between nocturia and non‐nocturia group before and after vaccination, and the changes in score.

Scores	Pre‐vaccinated			Post‐vaccinated			Change in scores		
	Nocturia (*n* = 273)	Non‐nocturia (*n* = 322)	*p*	Nocturia (*n* = 273)	Non‐nocturia (*n* = 322)	*p*	Nocturia (*n* = 273)	Non‐nocturia (*n* = 322)	*p*
ICSI									
ICSI‐Urgency, mean	0.62 ± 1.00	0.30 ± 0.70	< 0.001*	0.69 ± 1.08	0.39 ± 0.83	< 0.001*	0.07 ± 0.44	0.08 ± 0.50	0.714
ICSI‐Frequency, mean	1.58 ± 1.54	1.25 ± 1.30	0.004*	1.62 ± 1.34	1.34 ± 1.36	0.021*	0.04 ± 0.47	0.09 ± 0.53	0.174
ICSI‐Nocturia, mean	1.36 ± 0.81	0	< 0.001*	1.42 ± 0.88	0.12 ± 0.40	< 0.001*	0.07 ± 0.37	0.22 ± 0.70	0.066
ICSI‐bladder pain, mean	0.40 ± 0.83	0.25 ± 0.68	0.015*	0.43 ± 0.90	0.28 ± 0.74	0.026*	0.04 ± 0.41	0.04 ± 0.33	0.983
ICSI‐total score	3.96 ± 2.73	1.80 ± 1.95	< 0.001*	4.16 ± 2.98	2.14 ± 2.35	< 0.001*	0.21 ± 1.31	0.34 ± 1.34	0.235
ICPI									
ICPI‐Urgency, mean	0.80 ± 0.98	0.44 ± 0.85	< 0.001*	0.84 ± 1.00	0.48 ± 0.89	< 0.001*	0.04 ± 0.34	0.03 ± 0.45	0.696
ICPI‐Frequency, mean	0.71 ± 0.97	0.37 ± 0.74	< 0.001*	0.77 ± 1.01	0.43 ± 0.79	< 0.001*	0.06 ± 0.32	0.06 ± 0.40	0.877
ICPI‐Nocturia, mean	0.89 ± 0.95	0.22 ± 0.70	< 0.001*	0.96 ± 0.98	0.30 ± 0.78	< 0.001*	0.06 ± 0.36	0.08 ± 0.48	0.602
ICPI‐Bladder pain, mean	0.31 ± 0.78	0.15 ± 0.61	0.004*	0.32 ± 0.78	0.16 ± 0.59	0.004*	0.01 ± 0.32	0.01 ± 0.28	0.935
ICPI‐Total score, mean	2.18 ± 2.37	1.62 ± 2.01	0.002*	2.88 ± 3.04	1.35 ± 2.52	< 0.001*	0.70 ± 2.33	−0.27 ± 2.35	< 0.001*

*Note:* * indicates statistical significance *p* < 0.05.

These findings offer a sex‐specific perspective using IC/BPS‐relevant instruments [4] and suggest that COVID‐19 vaccination may trigger mild but measurable bladder sensory bother in a susceptible subgroup of women. The strong association with pre‐existing nocturia provides a pragmatic clinical signal for risk‐based counseling and short‐term post‐vaccination monitoring. Future prospective studies incorporating voiding diaries and inflammatory/metabolic biomarkers may clarify mechanisms and duration of these flares [3, 5].

## Funding

This research was funded by National Science and Technology Council of Taiwan (grant number: NSTC 114‐2314‐B‐037‐019), and Kaohsiung Medical University University Hospital (grant number: KMUH113‐3R53 and KMUH114‐4R58). The funders had no role in study design, data collection, analysis, and decision to publush.

## Conflicts of Interest

The authors declare no conflicts of interest.

## Supporting information


**Table S1:** Self‐reported bladder‐related and general adverse events following COVID‐19 vaccination (*N* = 595).
**Table S2:** Pre‐ and post‐vaccination ICSI/ICPI change of score. *, statistically significant between measurements (*p* < 0.05). ICPI, interstitial cystitis problem index; ICSI, interstitial cystitis symptoms index, SD, standard deviation.
**Table S3:** Univariable and multivariable analyses of factors associated with ICPI score deterioration after vaccination. *, statistically significant between measurements (*p* < 0.05). CI, confidence interval; ICPI, interstitial cystitis problem index; LUTS, lower urinary tract symptoms; OR, odds ratio, Ref: reference.
**Table S4:** Patient characteristics and distribution.
**Table S5:** Univariable logistic regression analysis of factors associated with ICSI Score deterioration after COVID‐19 vaccination.

## Data Availability

The data that support the findings of this study are available from the corresponding author upon reasonable request.
