# Supplementary material for: Pre‐Existing Nocturia Status Predicts Bladder Symptom Exacerbation Following COVID‐19 Vaccination in Women
Source: Kaohsiung J Med Sci. 2026 Jan 22;42(8):e70177. doi: 10.1002/kjm2.70177 (PMC13399632; doi:10.1002/kjm2.70177)
Supplement: Supplementary file 1 [file KJM2-42-e70177-s001.docx]

**Supplementary Table 1. Self-Reported Bladder-Related and General Adverse Events Following COVID-19 Vaccination (N = 595)**

| Adverse events | All  N=595 |
| --- | --- |
| **Bladder sensory symptoms** |  |
| Frequency, n (%) | 31 (5.2%) |
| De novo, n | 22 |
| Deterioration, n | 9 |
| Nocturia, n (%) | 34 (5.7%) |
| De novo, n | 25 |
| Deterioration, n | 9 |
| Urgency, no (%) | 23 (3.9%) |
| De novo, n | 15 |
| Deterioration, n | 8 |
| Bladder pain, n (%) | 11 (1.9%) |
| De novo, n | 9 |
| Deterioration, n | 2 |
| Seek medical help due to urinary-related symptoms, n (%) | 31 (5.2%) |
| **Generalized symptoms** |  |
| Headache, n (%) | 239 (40.2%) |
| Fever, n (%) | 263 (44.2%) |
| Fatigue, n (%) | 356 (59.8%) |
| Chills, n (%) | 151 (25.4%) |
| Sore arm/injected site pain, n (%) | 431 (72.4%) |
| **Musculoskeletal symptoms** |  |
| Arthritis/joint pains, n (%) | 90 (15.1%) |
| Muscle pain, n (%) | 260 (43.7%) |

**Supplementary Table 2. Pre- and post-vaccination ICSI / ICPI change of score.** *, statistically significant between measurements (p < 0.05). Abbreviation: ICPI: Interstitial cystitis problem index; ICSI: Interstitial cystitis symptoms index, SD: standard deviation

|  | ICSI | |  |  | ICPI | |  |
| --- | --- | --- | --- | --- | --- | --- | --- |
| Score | Pre-vaccination  (n=595) | Post-vaccination  (n=595) | *P*-value |  | Pre-vaccination  (n=595) | Post-vaccination  (n=595) | *P*-value |
| Frequency, mean ± SD | 1.40 ± 1.43 | 1.47 ± 1.46 | 0.001 |  | 0.53 ± 0.87 | 0.58 ± 0.91 | < 0.001 * |
| Nocturia, mean ± SD | 0.62 ± 0.87 | 0.72 ± 0.93 | < 0.001 * |  | 0.53 ± 0.89 | 0.60 ± 0.93 | < 0.001 * |
| Urgency, mean ± SD | 0.45 ± 0.86 | 0.53 ± 0.96 | < 0.001 * |  | 0.61 ± 0.93 | 0.64 ± 0.96 | 0.02 * |
| Bladder pain, mean ± SD | 0.31 ± 0.75 | 0.35 ± 0.82 | 0.01 |  | 0.22 ± 0.70 | 0.23 ± 0.69 | 0.492 |
| Total score, mean ± SD | 2.79 ± 2.57 | 3.07 ± 2.84 | < 0.001 * |  | 1.88 ± 2.20 | 2.06 ± 2.88 | 0.072 |

**Supplementary Table 3. Univariable and Multivariable Analyses of Factors Associated with ICPI Score Deterioration After Vaccination.** *, statistically significant between measurements (p < 0.05). Abbreviation: ICPI: Interstitial cystitis problem index; OR: Odds ratio, CI: Confidence interval, Ref: Reference, LUTS: Lower urinary tract symptoms

| Variables |  | ICPI Score Deterioration After Vaccination | | | | |
| --- | --- | --- | --- | --- | --- | --- |
|  |  | Crude OR  (95% CI) | *P*-value |  | Adjusted OR  (95% CI) | *P*-value |
| Age |  |  |  |  |  |  |
| 20-29 yr |  | Ref. |  |  | Ref. |  |
| 30-39 yr |  | 0.106 (0.012-0.966) | 0.047 * |  | 0.959 (0.541-1.701) | 0.887 |
| 40-49 yr |  | 0.156 (0.017-1.463) | 0.104 |  | 1.058 (0.642-1.745) | 0.824 |
| 50-59 yr |  | 0.184 (0.020-1.696) | 0.135 |  | 0.721 (0.408-1.271) | 0.258 |
| 60-69 yr |  | 0.153 (0.016-1.426) | 0.099 |  | 1.097 (0.412-2.919) | 0.853 |
| ≥ 70 yr |  | 0.333 (0.032-3.515) | 0.361 |  | 2.105 (0.207-21.407) | 0.529 |
| Comorbidity |  |  |  |  |  |  |
| Cardiovascular disease |  | 0.811 (0.278-2.365) | 0.701 |  | - | - |
| Hypertension |  | 2.818 (1.429-5.558) | 0.003 * |  | 1.537 (0.710-3.331) | 0.276 |
| Diabetes mellitus |  | 3.662 (0.906-14.793) | 0.068 |  | - | - |
| Hyperlipidemia |  | 5.435 (2.109-14.006) | <0.001 * |  | 3.420 (1.236-9.463) | 0.018 * |
| Pre-vaccination LUTS |  |  |  |  |  |  |
| Nocturia |  | 3.709 (2.604-5.282) | <0.001 * |  | 3.527 (2.389-5.206) | <0.001* |

**Supplementary Table 4. Patient characteristics and distribution**

| Patient Characteristics(n=595) | | | | |
| --- | --- | --- | --- | --- |
| Age | Number (n, %) |  | Comorbidity | Number (n, %) |
| 20-29 yr | 298 (50%) |  | Cardiovascular disease | 16 (2.7%) |
| 30-39 yr | 73 (12.2%) |  | Hypertension | 37 (6.2%) |
| 40-49 yr | 110 (18.5%) |  | Diabetes mellitus | 9 (1.5%) |
| 50-59 yr | 87 (14.6%) |  | Hyperlipidemia | 23 (3.9%) |
| 60-69 yr | 21 (3.5%) |  | Nocturia | 273 (45.9%) |
| ≥ 70 yr | 5 (0.8%) |  | Chronic kidney diseases | 3 (0.5%) |

**Supplementary Table 5. Univariable Logistic Regression Analysis of Factors Associated with ICSI Score Deterioration After COVID-19 Vaccination**

| Variables |  | ICSI Score Deterioration After Vaccination | |
| --- | --- | --- | --- |
|  |  | Crude OR  (95% CI) | *P*-value |
| Comorbidity |  |  |  |
| Cardiovascular disease |  | 1.006 (0.224-4.517) | 1.006 |
| Hypertension |  | 0.845 (0.291-2.457) | 0.757 |
| Diabetes mellitus |  | 0 (0.000-0.000) | 0.999 |
| Hyperlipidemia |  | 2.025 (0.729-5.628) | 0.176 |
| Pre-vaccination Nocturia |  | 0.642 (0.388-1.063) | 0.085 |
